# Bioactive glass nanoparticles induce pronounced cytotoxicity in human hepatocellular carcinoma Hep-G2 cells through ROS-mediated genomic instability and mitochondrial apoptosis

**DOI:** 10.1007/s00210-025-04731-6

**Published:** 2025-11-12

**Authors:** Hanan R. H. Mohamed, Aya A. Osman, Shahd Mosaad, Alaa H. Elsewedy, Habiba M. Zaki, Mayada E. Borai, Ahmed M. Aref, Gehan Safwat

**Affiliations:** 1 Department of Zoology, Faculty of Science, Cairo University, Giza, Egypt; 2 Faculty of Biotechnology, October University for Modern Sciences and Arts (MSA), 6th of October City, Egypt

**Keywords:** Bioactive glass nanoparticles, Hep-G2 cancer cells, MTT assay, ROS generation, Genomic instability, Mitochondrial dysfunction and apoptosis induction

## Abstract

Hepatocellular carcinoma (HCC) remains one of the leading causes of cancer-related mortality worldwide, emphasizing the urgent need for safer and more effective therapeutic strategies. Although bioactive glass nanoparticles (BGNPs) have been extensively studied in regenerative medicine, their direct anticancer potential, particularly against hepatic cancer has not been fully explored. To address this gap, the present study evaluated the therapeutic effects of BGNPs on Hep-G2 hepatic cancer cells, a widely used in vitro model for HCC compared to their impact on viability of normal HFB4 melanocytes. Cell viability was assessed using MTT cytotoxicity assay, while genomic stability was evaluated using the alkaline comet assay. The intracellular reactive oxygen species (ROS) level, mitochondrial membrane potential integrity, and apoptosis induction was also manifested in cancerous Hep-G2 cells to shed more light on BGNP anticancer potential. The results of MTT assay first revealed that BGNPs exhibit strong and selective cytotoxicity toward Hep-G2 hepatic cancer cells with an IC50 value of 72.77 μg/mL, compared to their minimal toxicity exhibited in normal HFB4 melanocytes with an IC50 value of 360.4 μg/mL. This strong targeted cytotoxicity of BGNPs toward Hep-G2 cancer cells was further confirmed by the calculated high selectivity index value of 4.95. Mechanistic studies demonstrated that BGNPs induce substantial (*p* < 0.001) genomic instability, mitochondrial dysfunction, and apoptosis in Hep-G2 cancer cells. These effects were driven by significantly (*p* < 0.001) elevated ROS production, marked (*p* < 0.001) loss of mitochondrial membrane potential, and remarkable dysregulation of critical genes, including significant (*p* < 0.001) upregulation of the pro-apoptotic p53 gene, alongside with marked (*p* < 0.001) downregulation of the anti-apoptotic Bcl-2 gene, and suppression of the ND3 gene involved in mitochondrial respiration. This study provides strong evidence that BGNPs exert selective and targeted cytotoxic effects on Hep-G2 hepatic cancer cells through a multifactorial mechanism involving oxidative stress, mitochondrial disruption, DNA damage, and apoptosis induction. Importantly, BGNPs exhibited minimal toxicity in normal HFB4 melanocytes, suggesting a favorable therapeutic index. To further validate these findings and explore the clinical applicability of BGNPS for hepatocellular carcinoma treatment, future studies including in vivo studies, targeted delivery strategies, and detailed analyses of DNA repair pathways, such as γ-H2AX foci formation and transcriptomic profiling are recommended.

## Introduction

Hepatocellular carcinoma (HCC) is the most prevalent form of primary liver cancer, accounting for approximately 75–85% of all liver cancer cases globally, and ranks as the third leading cause of cancer-related mortality worldwide (Sung et al. 2021; Tu et al. 2024). HCC poses a significant global health challenge, being the sixth most commonly diagnosed cancer. Its incidence is particularly high in regions with endemic hepatitis B and C virus infections, notably in Asia and sub-Saharan Africa. However, in Western countries, the rising incidence is increasingly linked to non-viral etiologies such as non-alcoholic fatty liver disease, obesity, excessive alcohol consumption, and metabolic syndromes (El-Serag and Kanwal 2014; Bray et al. 2018; Villanueva 2019; Llovet et al. 2021).

Despite advances in treatment, current therapeutic strategies for HCC, such as surgical resection, chemotherapy, and targeted therapies, remain limited by high recurrence rates, poor overall prognosis, and adverse marked systemic toxicity (Llovet et al. 2021). While systemic treatments such as chemotherapy and molecularly targeted agents are central to managing advanced stages of the disease, their overall clinical benefit remains modest. Conventional chemotherapeutic drugs like doxorubicin and cisplatin often exhibit poor tumor response and face challenges such as multidrug resistance and impaired tolerability due to the compromised hepatic function common in HCC patients (Hsu et al. 2014; Raoul et al. 2018; Girardi et al. 2023). Moreover, these therapies are associated with serious side effects, including hepatotoxicity, myelosuppression, gastrointestinal toxicity, and cardiotoxicity, which not only diminish patients’ quality of life but also limit long-term treatment adherence (Ghiaur et al. 2024; Famurewa et al. 2025). These limitations highlight the urgent need for novel, more selective therapeutic approaches that can effectively target tumor cells while minimizing damage to healthy tissues.

In recent years, nanotechnology has emerged as a transformative approach in cancer therapy, offering advantages such as targeted drug delivery, improved cellular uptake, and customizable physicochemical properties (Chehelgerdi et al. 2024; Tiwari et al. 2023). Among the various nanomaterials under investigation, bioactive glass nanoparticles (BGNPs) have attracted growing interest due to their exceptional biocompatibility, biodegradability, surface reactivity, and ability to interact with biological systems at both the molecular and cellular level in highly specific ways (Cannio et al. 2021; Drevet et al. 2024).

BGNPs are well recognized for their osteoconductive and osteoinductive properties and traditionally developed for orthopedic applications, particularly in bone tissue engineering and regenerative medicine (Hoppe et al. 2011; Jones 2013). Upon exposure to aqueous environments, these amorphous silicate-based materials release therapeutic ions, such as calcium (Ca^2+^), silicon (Si^4+^), sodium (Na^+^), and phosphate (P^5+^), in a controlled manner. This ion release contributes not only to their biological activity and regenerative potential but also enables modulation of key cellular signaling pathways, induction of oxidative stress, and disruption of mitochondrial function (Hoppe et al. 2011; Jones 2013; Pajares-Chamorro and Chatzistavrou; 2020; Drevet et al. 2024).

In oncology, bioactive glass has gained increasing attention for its potential to selectively target cancer cells; however, research to date has been confined to bulk and macroparticle forms. The direct cytotoxic potential of BGNPs against cancer cells has not yet been explored, even though the cytotoxic effects in particle systems largely based on several factors: particle size, surface area, ion release, and exposure duration (Baino et al. 2016; Kaou et al. 2023). For example, macroparticulate bioactive glass has demonstrated substantial cytotoxic activity against human osteosarcoma SaOS-2 cells and giant cell tumor of bone, while showing comparatively low toxicity toward normal bone-derived stromal cells, thereby highlighting its promise as a selective anticancer material (Fellenberg et al. 2022; Deliormanlı et al. 2024). Notably, when bioactive glass is engineered at the nanoscale, its physicochemical and biological properties undergo profound alterations, including enhanced cellular uptake, modified ion dissolution kinetics, and increased surface reactivity. These nanoscale-specific characteristics are expected to play a pivotal role in shaping, and potentially amplifying, the cytotoxic efficacy of BGNPs in cancer cells (Cannio et al. 2021; Drevet et al. 2024).

Despite these promising characteristics of BGNPs, research assessing their cytotoxicity, genotoxicity, and underlying mechanisms of action, particularly in cancer models, remains limited. The small number of studies available, coupled with inconsistencies in experimental conditions, highlights a significant gap in current knowledge and the need for more systematic and comprehensive investigations. Given that nanomaterials often exhibit distinct physicochemical and biological behaviors compared to their bulk or macroparticle counterparts, it is scientifically inappropriate to directly extrapolate findings from larger-scale materials to the nanoscale. This underscores the critical need for dedicated studies to evaluate the therapeutic potential, selectivity, and biosafety of BGNPs in oncological applications (Cannio et al. 2021; Deliormanlı et al. 2024; Drevet et al. 2024).

Based on the abovementioned promising properties of BGNPs and the complete lack of data regarding their therapeutic potential against HCC, this study estimated for the first time the direct cytotoxic effect of BGNPs on human normal HFB4 melanocytes and hepatic cancer Hep-G2 cells, alongside with assessing the genomic DNA integrity, mitochondrial membrane potential, oxidative stress level, and apoptosis induction in Hep-G2 cells, independent of any chemotherapeutic drug loading. Unlike previous studies that primarily focus on the regenerative applications of BGNPs or their use as passive drug carriers, this work explores their direct cytotoxic potential through reactive oxygen species (ROS)-mediated oxidative stress, mitochondrial dysfunction, and genomic instability. By comparing the effects of BGNPs on cancerous Hep-G2 cells versus normal HFB4 melanocytes, this study also highlights their selective toxicity and potential as a standalone nanotherapeutic agent. The findings offer novel mechanistic insights into the ROS-mitochondria-DNA damage axis in liver cancer treatment and lay the groundwork for future translational research in nanomedicine for hepatocellular carcinoma.

## Materials and methods

### Chemicals

The BGNPs utilized in this study were purchased in fine white powder form from Nanotech Company, 6th October City, Cairo, Egypt. For experimental use, BGNPs were accurately weighed using a high-precision analytical balance and suspended in dimethyl sulfoxide (DMSO; CAS No. 67–68-5, Sigma-Aldrich, St. Louis, MO, USA; Cat. No. D2650) to prepare stock solutions. The suspensions were sonicated in a bath sonicator for 15–20 min immediately before use to ensure uniform dispersion and to minimize particle agglomeration. Several analytical and molecular-grade reagents were also employed. 3-(4,5-Dimethylthiazol-2-yl)−2,5-diphenyl tetrazolium bromide (MTT; Sigma-Aldrich, Cat. No. M5655) and trypan blue dye (Sigma-Aldrich, Cat. No. T6146) were obtained for cytotoxicity and viability assays. Cell culture reagents, including Dulbecco’s Modified Eagle Medium (DMEM; Lonza, Belgium; Cat. No. 12-604F), HEPES buffer solution (Lonza, Cat. No. 17-737E), l-glutamine (Lonza, Cat. No. 17-605E), gentamycin (Lonza, Cat. No. 17-518Z), and 0.25% Trypsin–EDTA (Lonza, Cat. No. BE02-007E), were used for maintaining Hep-G2 and HFB4 cells. Media were supplemented with 10% fetal bovine serum (FBS; Lonza, Cat. No. 14-801F) and 1% gentamycin to support cell growth and sterility. Phenol red-free formulations were used to eliminate optical interference during spectrophotometric assays. All reagents were freshly prepared as required, and all culture/treatment procedures were performed aseptically in a Class II laminar Flow biosafety cabinet to ensure sterility and reproducibility.

### Characterization of BGNPs

The BGNPs were thoroughly characterized using a combination of advanced physicochemical techniques to confirm their structural, morphological, and colloidal properties. X-ray diffraction (XRD) analysis was conducted using a charge-coupled device diffractometer (XPERT-PRO, PANalytical, Almelo, Netherlands) to determine the crystalline or amorphous nature of the BGNPs. The XRD patterns were recorded over an appropriate 2θ range with Cu Kα radiation, enabling identification of characteristic peaks and verification of phase composition.

To assess the hydrodynamic particle size distribution and zeta potential, dynamic light scattering (DLS) measurements were performed using a Malvern Zetasizer Nano Series (Malvern Instruments, Westborough, MA, USA). The instrument utilizes a helium–neon laser (*λ* = 633 nm, maximum output 5 mW) and backscatter detection at a fixed angle (173°) to evaluate particle dispersion stability and surface charge in aqueous media, both of which are critical parameters for predicting nanoparticle-cell interactions. For detailed analysis of particle morphology and size at the nanoscale, transmission electron microscopy (TEM) was carried out using a Tecnai G20 Super Twin microscope (FEI, USA), equipped with a double-tilt specimen holder and operating at an accelerating voltage of 200 kV. Samples for TEM imaging were prepared by dropping a small volume of the BGNPS suspension onto a carbon-coated copper grid and allowing it to air-dry. TEM images provided high-resolution visualization of particle shape, aggregation state, and size distribution, complementing the DLS measurements and confirming the nanoscale nature of the BGNPs.

### Cell culture and propagation

Human HFB4 normal melanocytes and Hep-G2 hepatocellular carcinoma cells were kindly provided by the Regional Center for Mycology and Biotechnology, Al-Azhar University (Cairo, Egypt), a certified cell culture supplier that maintains authenticated and quality-controlled cell lines. According to the center’s standard procedures, cell line identity is regularly confirmed by short tandem repeat profiling and screened for mycoplasma contamination prior to distribution. Each cell line was cultured independently in DMEM (high glucose, 4.5 g/L) supplemented with 10% heat-inactivated FBS and 50 μg/mL gentamycin. To further ensure sterility, the medium was additionally supplemented with 100 U/mL penicillin and 100 μg/mL streptomycin. All cultures were maintained at 37 °C in a humidified 5% CO₂ atmosphere, with media changed every 2–3 days. Subculturing was performed using 0.25% trypsin–EDTA when cells reached 70–80% confluence. Only exponentially growing cultures with ≥ 90% viability were used in subsequent assays to ensure reproducibility and reliability of the data.

### Estimation of BGNPs effect on HFB4 and Hep-G2 cell viability

To assess the cytotoxic effect of BGNPs on human HFB4 normal melanocytes and Hep-G2 hepatocellular carcinoma cells, the 3-(4,5-dimethylthiazol-2-yl)−2,5-diphenyltetrazolium bromide (MTT) assay was conducted based on the previously established protocols by Mosmann (1983), El-Zahabi et al. (2019), and Abdelsalam et al. (2022), with slight modifications to suit experimental conditions. Both HFB4 and Hep-G2 cells were harvested and seeded into sterile, flat-bottom 96-well tissue culture plates (Falcon, NJ, USA) at a density of 1 × 10^4^ cells per well in 100 μL of complete DMEM growth medium containing 10% heat-inactivated FBS, 1% l-glutamine, and antibiotics (100 U/mL penicillin and 100 μg/mL streptomycin). After seeding, the plates were incubated for 24 h at 37 °C in a humidified atmosphere containing 5% CO₂ to ensure proper cell adhesion and recovery.

After the initial incubation, the culture medium was aspirated and replaced with fresh medium containing serial two-fold dilutions of BGNPs at concentrations of 7.8, 15.6, 31.25, 62.5, 125, 250, 500, and 1000 μg/mL. Each concentration was tested in triplicate. Control wells received only the complete growth medium without BGNPs. The plates were then incubated for an additional 72 h under the same conditions to allow for nanoparticle exposure and cytotoxic response. Following the 72-h treatment period, the medium was carefully removed, and 100 μL of phenol red-free complete DMEM was added to each well. Subsequently, 10 μL of MTT stock solution (12 mM), prepared by dissolving 5 mg of MTT in 1 mL of phosphate-buffered saline (PBS), was added to each well, including controls. The plates were then incubated for 4 h at 37 °C in the dark to allow viable cells to enzymatically reduce the MTT into insoluble purple formazan crystals. After incubation, 85 μL of the medium was gently aspirated without disturbing the formazan crystals. Then, 50 μL of DMSO was added to each well to dissolve the crystals, followed by gentle shaking or pipetting. The plates were incubated at 37 °C for 10 min to ensure complete solubilization. The absorbance of each well was measured at 590 nm using a microplate reader (SunRise, TECAN, Inc., USA). Cell viability was expressed as a percentage relative to the untreated control group and calculated using the formula: cell viability (%) = (ODt/ODc) × 100.

Where ODt is the mean optical density of treated wells and ODc is the mean optical density of control wells. The half-maximal inhibitory concentration (IC50) values were calculated using non-linear regression analysis with GraphPad Prism software (San Diego, CA, USA), based on data obtained from three independent experiments. To evaluate the selectivity of BGNPs, the Selectivity Index (SI) was calculated as the ratio of IC50 values between normal HFB4 melanocytes and Hep-G2 cancer cells: An SI greater than 1 indicates selective cytotoxicity toward cancer cells over normal cells. All data were reported as mean ± standard deviation (SD).

### BGNPs treatment and preparation of Hep-G2 cells for molecular analysis

Human Hep-G2 hepatocellular carcinoma cells were cultured in T25 flasks using DMEM supplemented with 10% heat-inactivated FBS, 1% l-glutamine, and antibiotics (100 U/mL penicillin and 100 μg/m streptomycin). Cells were maintained under standard conditions at 37 °C in a humidified atmosphere containing 5% CO₂ until they reached approximately 70–80% confluence. At this point, the cultures were divided into untreated control and BGNP-treated Hep-G2 cells. Control cells were treated with DMSO at a concentration of less than 0.1% (v/v), while the treated cells were exposed to BGNPs at the IC50 concentration determined by MTT assay. Both control and treated cells were incubated for 72 h under the same standard conditions.

Following the treatment period, all cells were harvested by enzymatic detachment using 0.25% trypsin–EDTA. The cells were then collected by centrifugation at 1500 rpm for 5 min at 4 °C. The resulting cell pellets were washed twice with ice-cold PBS (pH 7.4) to remove any residual media and treatment compounds. After the final wash, the pellets were resuspended in PBS and stored at – 80 °C for subsequent molecular and biochemical analyses. All treatments were conducted in triplicate to ensure data reproducibility and allow for statistical validation.

### Estimation of Hep-G2 genomic stability using Alkaline Comet Assay

The alkaline single-cell comet assay was performed to quantitatively assess DNA strand breaks and genomic instability in Hep-G2 cells following exposure to the IC50 concentration of BGNPs for 72 h. The assay was conducted following the standardized protocols described by Tice et al. (2000) and Langie et al. (2015). Briefly, 15 μL of a Hep-G2 cell suspension containing about 10,000 cells was gently mixed with 60 μ of 0.5% low-melting-point agarose (prepared in PBS and maintained at 37 °C) and immediately spread onto microscope slides pre-coated with 1% normal-melting-point agarose. Slides were allowed to solidify at room temperature for 30 min in a horizontal position.

After solidification, slides were immersed in cold, freshly prepared lysis buffer (2.5 M NaCl, 100 mM EDTA, 10 mM Tris–HCl, pH 10) freshly supplemented with 1% Triton X-100 and 10% DMSO to disrupt membranes and remove nuclear proteins. Lysis was carried out at 4 °C for 24 h in complete darkness to prevent additional DNA damage. Slides were then transferred to an electrophoresis tank containing freshly prepared alkaline buffer (300 mM NaOH, 1 mM EDTA, pH > 12) and incubated for 15 min to allow DNA unwinding and expression of alkali-labile sites.

Electrophoresis was conducted at 25 V and 300 mA for 30 min at 4 °C to maintain consistent temperature and reproducibility. Following electrophoresis, slides were neutralized with 0.4 M Tris–HCl buffer (pH 7.5) for 5 min, fixed in cold absolute ethanol for 5 min, and air-dried. Dried slides were stained with 50 μL of ethidium bromide (20 μg/mL) and visualized using a fluorescence microscope. For each sample, 50 randomly selected comets were captured and analyzed using COMETSCORE TM software. DNA damage was quantified based on tail length, %DNA in tail, and tail moment. Results are presented as mean ± standard deviation (SD) from three independent experiments, with statistical analysis performed to evaluate the genotoxic effects of BGNPs.

### Assessment of Hep-G2 ROS generation using 2,7-DCFH-DA fluorescent probe

The intracellular production of ROS in Hep-G2 hepatocellular carcinoma cells following exposure to BGNPs was quantitatively measured using the cell-permeable fluorescent probe 2,7-dichlorofluorescin diacetate (2,7-DCFH-DA), following the method described by Siddiqui et al. (2010) with slight modifications. After treating Hep-G2 cells with BGNPs at the IC50 concentration for 72 h, the cells were harvested and were then resuspended in PBS to achieve a uniform suspension at a concentration of approximately 1 × 10^6^ cells/mL. For ROS detection, equal volumes of the Hep-G2 cell suspension and 20 μM 2,7-DCFH-DA solution were gently mixed in sterile microcentrifuge tubes. The mixture was incubated in the dark at room temperature for 30 min to allow sufficient uptake of the dye and its intracellular deacetylation by cellular esterases. During this incubation period, the non-fluorescent 2,7-DCFH-DA freely diffused across the cell membrane and was enzymatically converted into non-fluorescent 2,7-dichlorofluorescin (DCFH). In the presence of ROS, DCFH was rapidly oxidized to the highly fluorescent compound 2,7-dichlorofluorescein (DCF), with the fluorescence intensity directly correlating to the intracellular ROS levels.

Following the incubation period, the stained cells were gently pipetted onto clean, pre-labeled glass microscope slides to form a thin uniform layer. The slides were examined under an epifluorescence microscope equipped with appropriate filters for detecting DCF fluorescence. Images were captured at 200 × magnification from randomly selected fields, ensuring consistent exposure settings across all samples. The green fluorescence intensity emitted by the oxidized probe (DCF) served as a quantitative indicator of intracellular ROS production. Fluorescence images were analyzed using Image Fiji software, and the ROS level in BGNPS-treated Hep-G2 cells was statistically compared to those in untreated control cells by analyzing the relative fluorescence intensity, thereby enabling evaluation of oxidative stress induction by the nanoparticles. All experiments were conducted in triplicate to ensure data reliability and statistical robustness.

### Analysis of Hep-G2 mitochondrial membrane potential using Rhodamine-123 staining

The mitochondrial membrane potential, a key indicator of mitochondrial integrity and early-stage apoptosis, was assessed in Hep-G2 hepatocellular carcinoma cells following treatment BGNPs at their predetermined IC50 concentration. This assessment was conducted based on the protocol described by Zhang et al. (2011) using Rhodamine-123, a cell-permeable, cationic fluorescent dye that selectively accumulates in the mitochondria of viable cells with intact membrane polarization. Hep-G2 cells were cultured under standard conditions and treated with BGNPs for 72 h at the IC₅₀ concentration determined from prior MTT cytotoxicity analysis. At the end of the treatment period, both BGNPS-treated and -untreated (control) cells were harvested and resuspended in PBS to obtain a uniform cell suspension at an approximate density of 1 × 10^6^ cells/mL.

For staining, equal volumes of Rhodamine-123 working solution (10 μg/mL) and the cell suspension were gently mixed in sterile, light-protected microcentrifuge tubes. The mixtures were incubated at 37 °C for 1 h in the dark to ensure efficient dye uptake by metabolically active mitochondria, while minimizing photobleaching. After incubation, the cells were washed twice with cold PBS to remove unbound dye and reduce nonspecific fluorescence. Subsequently, a small aliquot of the stained suspension was carefully dispensed onto pre-cleaned, labeled glass microscope slides, spread into a thin, even monolayer and immediately covered with sterile coverslips to preserve sample integrity and fluorescence signal. The prepared slides were examined using an epifluorescence microscope equipped with the appropriate filter set at 200 × magnification. Fluorescent micrographs were acquired from multiple randomly selected fields for all cells, maintaining consistent exposure settings to ensure reliable comparison. The fluorescence intensity of Rhodamine-123, reflecting the extent of mitochondrial polarization, was quantified using Fiji (ImageJ) software, and the mean fluorescence intensity per cell was calculated for both control and BGNPS-treated cells. A notable decrease in Rhodamine-123 fluorescence in the treated cells indicated loss of mitochondrial membrane potential, a characteristic marker of early mitochondrial dysfunction and apoptosis initiation. All experimental treatments were conducted in triplicates, and data were expressed as mean ± SD to ensure reproducibility and statistical reliability.

### Estimation of apoptosis induction in Hep-G2 cells using chromatin diffusion assay

Apoptosis in both untreated (control) and BGNP-treated Hep-G2 hepatic cancer cells was assessed using the chromatin diffusion assay based on the principle that apoptotic cells contain numerous alkali-labile sites. Under alkaline conditions, these sites lead to the fragmentation of DNA into smaller pieces, which can diffuse within an agarose matrix. This diffusion creates a halo-like structure with a hazy outline surrounding the nucleus, distinguishing apoptotic cells from intact ones (Singh 2000). To perform the assay, microgel electrophoresis slides were first pre-coated with a thin layer of 0.7% agarose. A mixture of Hep-G2 cells and low-melting-point agarose was then gently layered onto these pre-coated slides and spread evenly to embed the cells within the gel. The slides were left to air-dry at room temperature, allowing the agarose to solidify and immobilize the cells within the matrix.

Once dried, the slides were immersed in a cold lysis buffer for 10 min, which lysed all cellular components except for the nuclear DNA. This step ensured that only the DNA remained embedded in the agarose. Following lysis, the slides were neutralized using a freshly prepared Tris buffer to halt the alkaline reaction, and then fixed in cold absolute ethanol to preserve the structural integrity of the diffused DNA. Before microscopic examination, the slides were stained with ethidium bromide, a fluorescent dye that binds specifically to DNA. The stained slides were then observed under a fluorescence microscope, and cells were examined for the presence of diffuse DNA halos, a hallmark of apoptotic cells. A total of 1000 cells per sample were evaluated, and the percentage of apoptotic cells (those exhibiting diffuse halos with a hazy boundary) was calculated.

### Detection of apoptosis induction in Hep-G2 cells using DAPI staining

To estimate apoptosis induction in Hep-G2 hepatocellular carcinoma cells, a nuclear staining assay using 4′,6-diamidino-2-phenylindole (DAPI) was conducted to detect characteristic apoptotic changes such as chromatin condensation and nuclear fragmentation (Guan and Guan 2020). Hep-G2 cells were initially seeded at a density of 1 × 10^4^ cells per well in sterile, flat-bottom 96-well plates containing complete DMEM and treated with the IC50 concentration of BGNPs for 72 h. After treatment, the culture medium was removed, and cells were washed twice with PBS to remove residual compounds. Fixation was performed with 4% paraformaldehyde for 15 min at room temperature, followed by another PBS wash. Cells were then stained with DAPI (1 μg/mL in PBS) for 1 h in the dark to allow nuclear DNA binding. Excess stain was removed with a gentle PBS rinse, and cells were immediately observed under a fluorescence microscope with a DAPI filter. Images were captured at 200 × magnification from multiple randomly selected fields using consistent exposure settings. Apoptotic cells were identified by distinct nuclear morphology, including bright, condensed chromatin, nuclear margination, and apoptotic body formation, in contrast to the uniform, less intense staining in non-apoptotic cells. Quantification was performed by counting 1000 cells per treatment, and the percentage of apoptotic nuclei was calculated. All experiments were conducted in triplicate, and data were reported as mean ± SD to ensure accuracy and reproducibility.

### Quantification of p53, ND3, and Bcl2 gene expression in Hep-G2 cells

To estimate the effect of BGNPs on apoptosis induction and mitochondrial function, the mRNA expression level of p53 (a pro-apoptotic gene), ND3 (a mitochondrial gene involved in electron transport), and Bcl2 (an anti-apoptotic gene) were measured in Hep-G2 hepatic cancer cells after 72 h of exposure to the IC50 concentration of BGNPs using quantitative real-time PCR (qRT-PCR). Total RNA was extracted from both untreated control and treated Hep-G2 cells using the GeneJET RNA Purification Kit (Thermo Fisher Scientific, USA), following the manufacturer’s protocol. The purity and concentration of RNA were assessed using a NanoDrop spectrophotometer. On 1 μg of total RNA from each sample was reverse transcribed into complementary DNA (cDNA) using the High-Capacity cDNA Reverse Transcription Kit (Applied Biosystems, USA). qRT-PCR was carried out using SYBR Green PCR Master Mix and gene-specific primers listed in Table 1 (Suzuki et al. 1999; Lai et al. 2013; Grzybowska-Szatkowska and Ślaska 2014). Reactions were performed in triplicate on a StepOnePlus Real-Time PCR System (Applied Biosystems) under optimized conditions. GAPDH was used as an internal control to normalize gene expression level. Fold changes in the expression of tested p53, ND3, and Bcl2 genes were calculated using the comparative Ct (ΔΔCt) method. Results are expressed as mean ± SD from three independent experiments, providing insight into how BGNPs regulate apoptotic and mitochondrial gene expression in Hep-G2 hepatic cancer cells.

**Table 1:** Sequences of primers used in qRT-PCR

Gene	Strand	Primer’s sequences
**GAPDH**	**Forward**	**5′-GAAGGTGAAGGTCGGAGTCA-3′**
	**Reverse**	**5′-GAAGATGGTGATGGGATTTC-3′**
**ND3**	**Forward**	**5′-CGCCGCCTGATACTGGCAT-3′**
	**Reverse**	**5′-CTAGTATTCCTAGAAGTGAG-3′**
**BCL-2**	**Forward**	**5′-TCCGATCAGGAAGGCTAGAGT-3′**
	**Reverse**	**5′-TCGGTCTCCTAAAAGCAGGC-3′**
**P53**	**Forward**	**5′-CAGCCAAGTCTGTGACTTGCACGTAC-3′**
	**Reverse**	**5′-CTATGTCGAAAAGTGTTTCTGTCATC-3′**

### Statistical analysis

All experimental data were statistically analyzed using the Statistical Package for the Social Sciences (SPSS). Results are presented as the mean ± SD derived from the conducted experiments, each performed in triplicate to ensure reproducibility and reliability. To evaluate the significance of differences in gene expression level, apoptotic cell counts, and other measured parameters between BGNP-treated and untreated Hep-G2 hepatic cancer cells, an unpaired two-tailed Student’s *t*-test was applied. A *p*-value less than 0.05 (*p* < 0.05) was considered statistically significant.

## Results

### Characterization of BGNPs

**Fig. 1:**
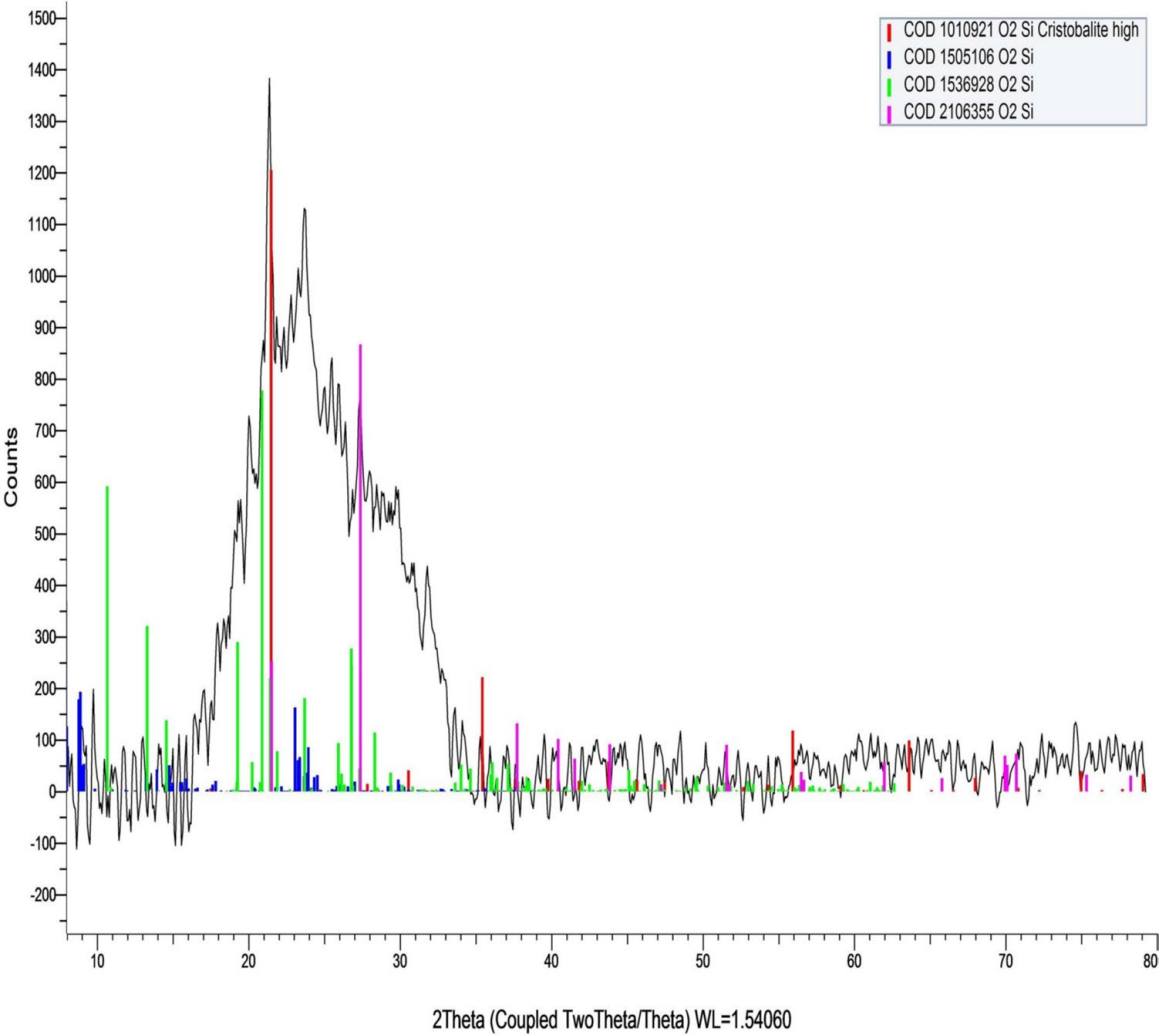
XRD analysis showed a broad diffuse spectrum, indication the amorphous nature of the BGNPs

**Fig. 2:**
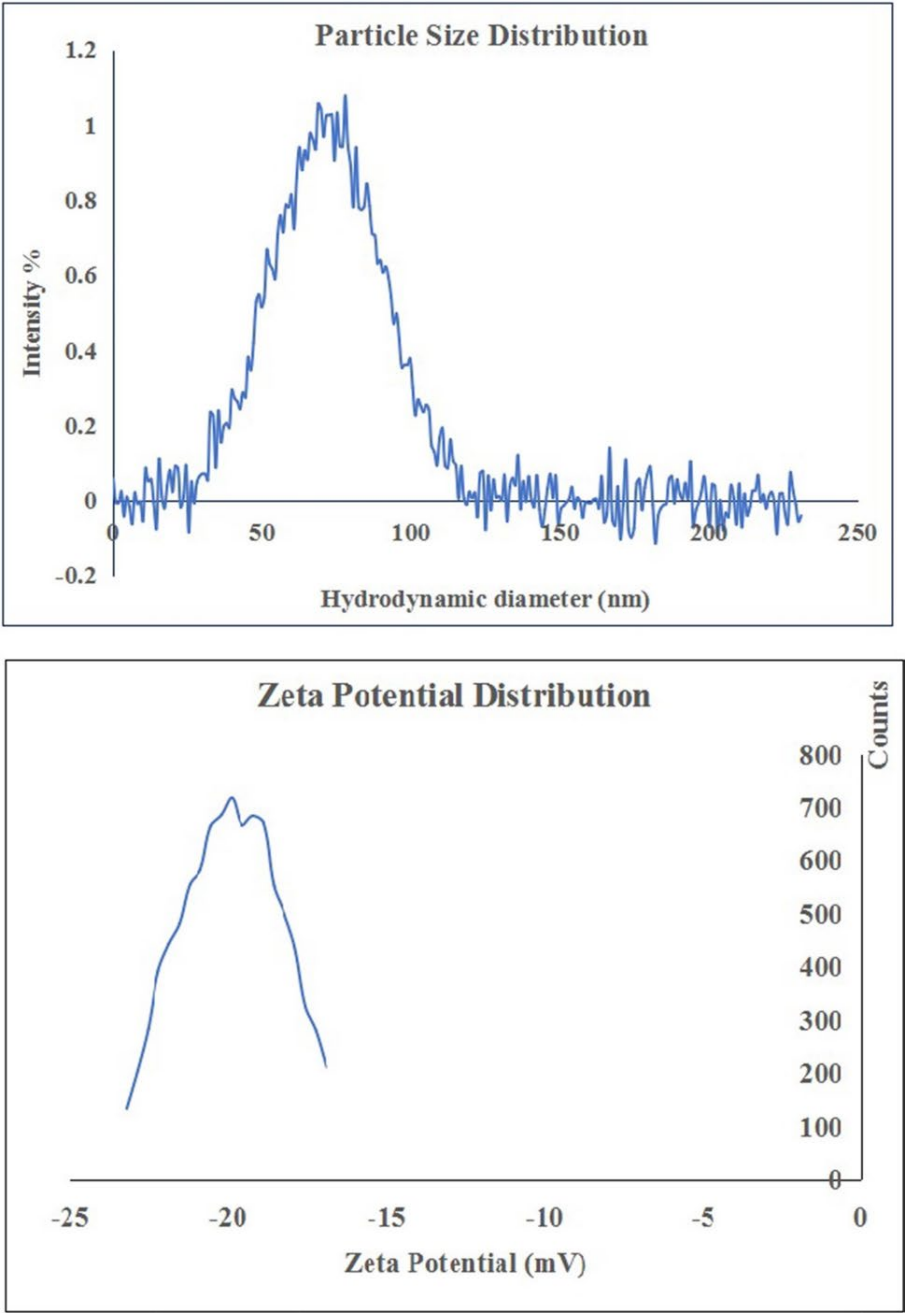
DLS analysis revealed that the BGNPs suspension exhibited a uniform particle size distribution with an average particle size of 73.3 nm and a zeta potential of − 19.89 mV

**Fig. 3:**
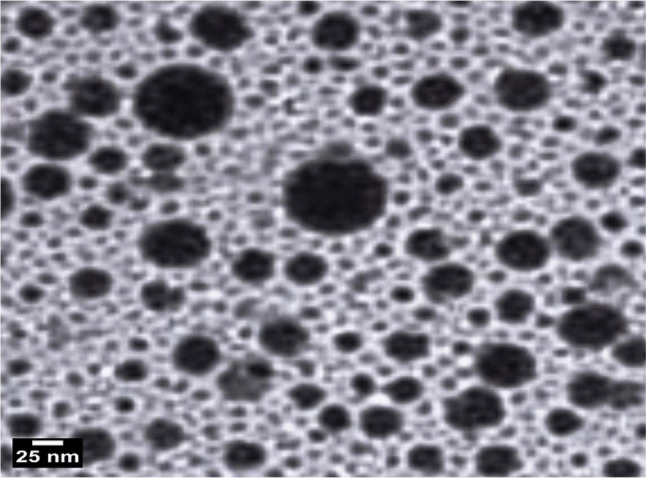
TEM analysis revealed that the BGNPs possess a spherical morphology with an average particle size of 29.5 nm

As depicted in Fig. 1 characterization of BGNPs using XRD analysis exhibited a broad diffuse spectrum spanning the diffraction angle (2θ) range of 15.48° to 35.11°. which is characteristic of an amorphous glass structure. This broad feature indicates an amorphous structure, characterized by the absence of long-range crystalline order. This pattern is typical of glassy materials and is often associated with enhanced bioactivity due to increased surface area and reactivity. The results of DLS analysis showed a particle size range of approximately 50 to 100 nm, with an average hydrodynamic diameter of 73.3 nm (Fig. 2). The polydispersity index (PDI) was found to be less than 0.3 (0.27), indicating a relatively narrow and homogeneous size distribution. The surface charge of BGNPs, measured by zeta potential analysis, was determined to be − 19.89 mV, suggesting moderate stability of the nanoparticle suspension and a negatively charged surface (Fig. 2). Further morphological characterization using TEM confirmed that the BGNPs were predominantly spherical with smooth surfaces. The particles appeared well-dispersed, with minimal signs of agglomeration, reflecting effective synthesis and stabilization conditions. The average particle size determined from TEM images was approximately 29.5 nm, corroborating the nanoscale nature of BGNPs (Fig. 3).

### BGNPs induce potent preferential cytotoxicity toward Hep-G2 hepatic cancer cells

**Fig. 4:**
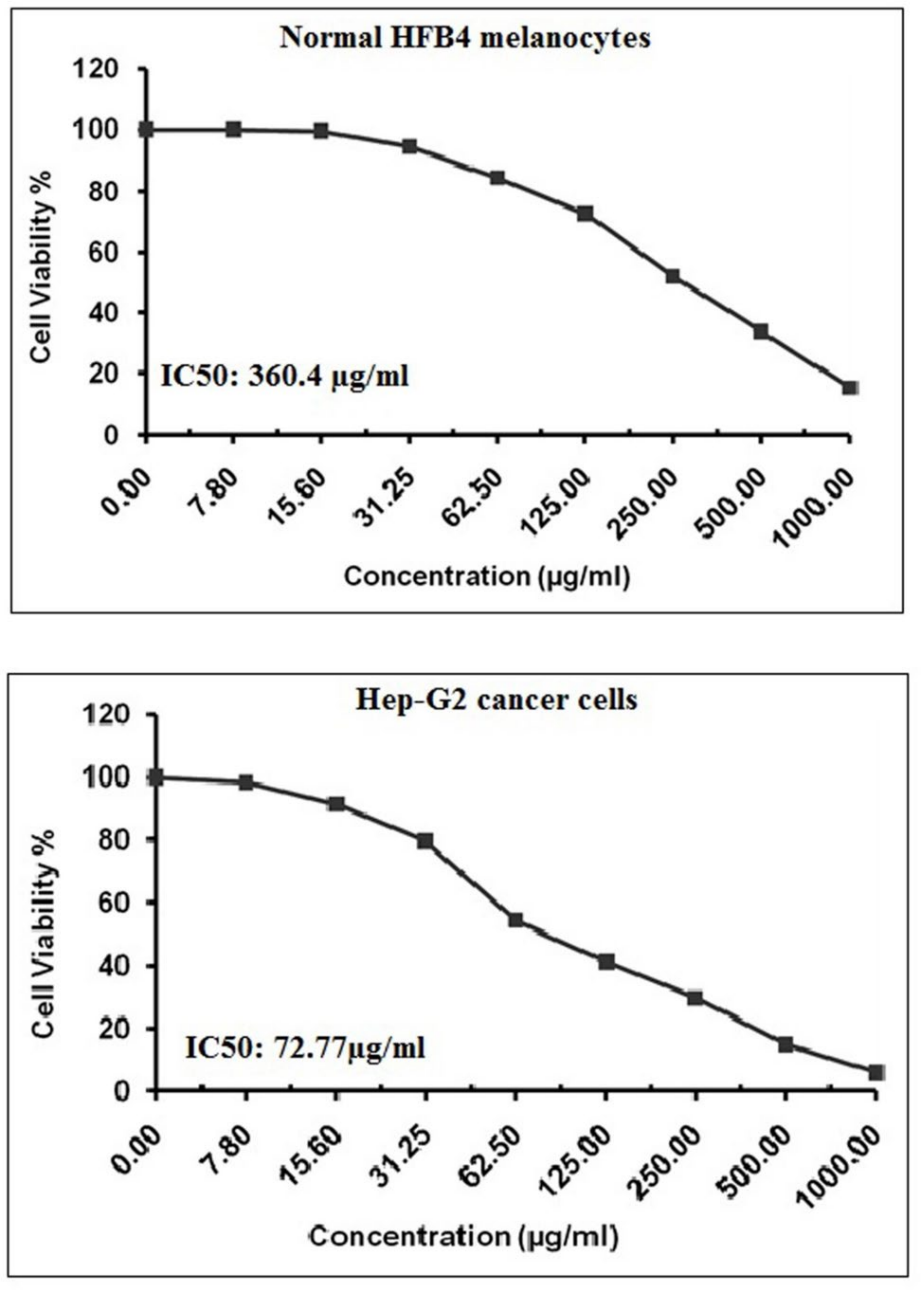
Viability profile of human normal HFB4 melanocytes and Hep-G2 cancer cells following 72-h exposure to BGNPs at twofold increasing concentrations (7.8, 15.6, 31.25, 62.5, 125, 250, 500, and 1000 μg/mL), assessed using the MTT assay

Evaluation of BGNP cytotoxicity using the MTT assay demonstrated a strong concentration-dependent reduction in Hep-G2 cells viability upon exposure to increasing concentrations (7.8, 15.6, 31.25, 62.5, 125, 250, 500, and 1000 μg/mL) of BGNPs for 72 h as illustrated in Fig. 4. The IC50 value of BGNPS was determined to be 72.77 μg/mL, indicating a significant cytotoxic effect against the cancerous Hep-G2 cell line. Conversely, the viability of normal HFB4 melanocytes was only minimally affected by BGNP exposure across the same concentration range and treatment duration. The IC50 value for normal HFB4 melanocytes was considerably higher, measured at 360.4 μg/mL, suggesting that BGNPs have selective cytotoxicity with considerably lower toxicity toward normal HFB4 cells (Fig. 4). This differential cytotoxic response implies selective targeting of Hep-G2 cancer cells by the BGNPs, sparing normal HFB4 cells from substantial damage. Furthermore, the calculated selectivity index was found to be 4.95, and this high SI value reinforces the strong and preferential cytotoxicity of BGNPs against Hep-G2 cancer cells. Consequently, more molecular studies were carried out on Hep-G2 cancer cells treated with the IC50 concentration of BGNPS to further explore their therapeutic potential against hepatocellular carcinoma.

### BGNPs cause dramatic genomic DNA in Hep-G2 cancer cells

**Fig. 5:**
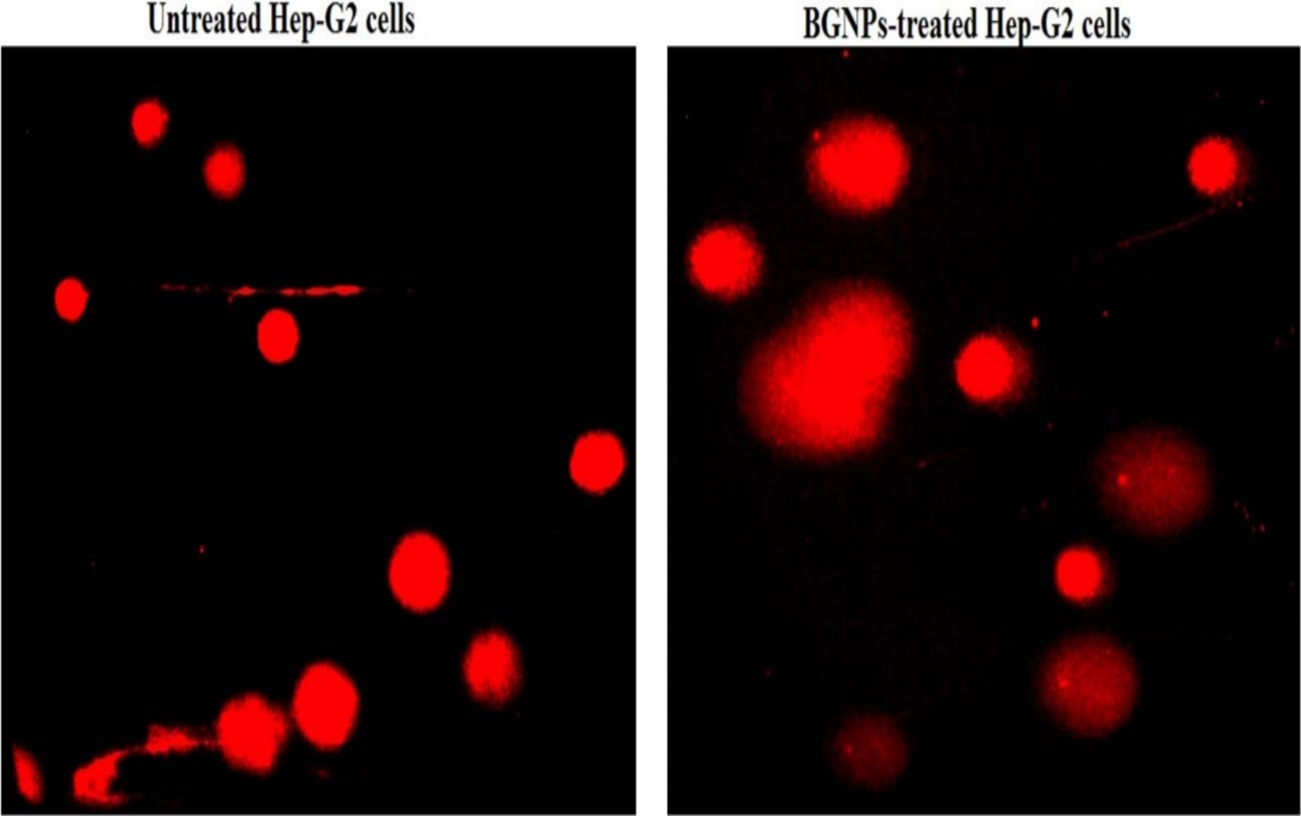
Examples for the scored Comet nuclei with intact DNA in untreated Hep-G2 cells and those with damaged DNA in Hep-G2 cancer cells treated with the IC50 concentration of BGNPs (72.77 μg/mL) for 72 h. Magnification 200 ×

Estimation of genomic DNA integrity using alkaline single-cell Comet assay demonstrated a pronounced induction of genomic DNA damage in Hep-G2 hepatic cancer cells treated with the IC50 concentration (72.77 μg/mL) of BGNPs for 72 h. This dramatic damage was quantitatively reflected by statistically significant elevations (*p* < 0.001) in key DNA damage-indicative parameters: tail length, %DNA in tail, and tail moment, in BGNP-treated Hep-G2 hepatic cancer cells when compared to untreated control cells, as presented in Table 2 and illustrated in Fig. 5. These parameters are widely recognized as sensitive indicators of DNA strand breaks and fragmentation, thereby confirming the genotoxic potential of BGNPs under the applied experimental conditions. Furthermore, visual analysis of representative Comet images in Fig. 5 supports the quantitative findings. The untreated control cells exhibited compact, undisturbed Comet nuclei, indicative of intact genomic DNA, whereas BGNP-treated Hep-G2 cells displayed extensive DNA migration from the nucleus, forming distinct comet-like tails that signify substantial DNA fragmentation.

**Table 2:** Genomic DNA integrity in Hep-G2 human hepatocellular carcinoma cells after 72-h exposure to the IC50 concentration (72.77 μg/mL) of BGNPs

	Treatment (concentration)	DNA integrity markers		
		Tail length (px)	%DNA in tail	Tail moment
Hep-G2 cancer cells	Untreated (0.00 μg/mL)	4.41 ± 1.36	22.92 ± 2.66	1.04 ± 0.44
	BGNP treated (72.77 μg/mL)	17.93 ± 2.36^***^	40.65 ± 1.11^***^	7.34 ± 0.92^***^

Results are expressed as mean ± SD

^***^Indicates statistical significant difference from the compared untreated Hep-G2 control cells at *p* < 0.001, using independent Student *t*-test

### BGNPs excessively generate ROS within Hep-G2 cancer cells

**Fig. 6:**
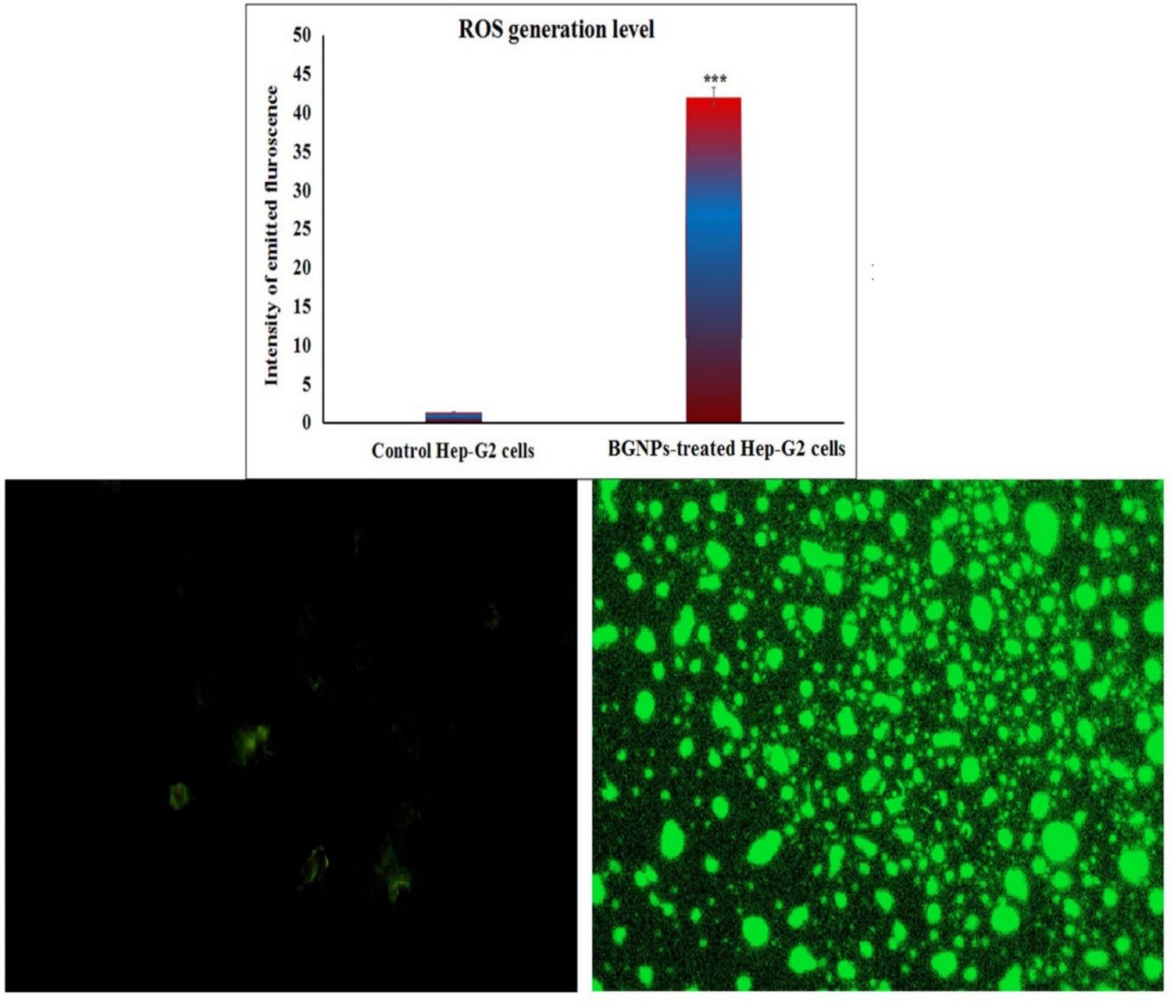
Production level of ROS generation within the untreated and treated Hep-G2 hepatic cancer cells with the IC50 concentration (72.77 μg/mL) of BGNPs for 72 h. Magnification 200 ×

As illustrated in Fig. 6, exposure of Hep-G2 hepatic cancer cells to BGNPs at the IC50 concentration (72.77 μg/mL) for 72 h resulted in a significant increase in intracellular ROS generation level. This remarkable increase was quantitatively confirmed by a highly significant rise (*p* < 0.001) in the fluorescence intensity of 2,7-DCFH-DA, a ROS-sensitive fluorescent probe. The dye becomes fluorescent upon oxidation by ROS, making it a reliable indicator of oxidative stress. Compared to untreated control cells, BGNP-treated Hep-G2 hepatic cancer cells exhibited markedly stronger fluorescence signals, indicating a substantial buildup of ROS within the cytoplasm. These results suggest that BGNPs induce oxidative stress in Hep-G2 cells and support the hypothesis that ROS generation plays a central role in the cytotoxic effects exerted by BGNPs in Hep-G2 hepatic cancer cells.

### BGNPs dramatically disrupt mitochondrial membrane integrity in Hep-G2 hepatic cancer cells

**Fig. 7:**
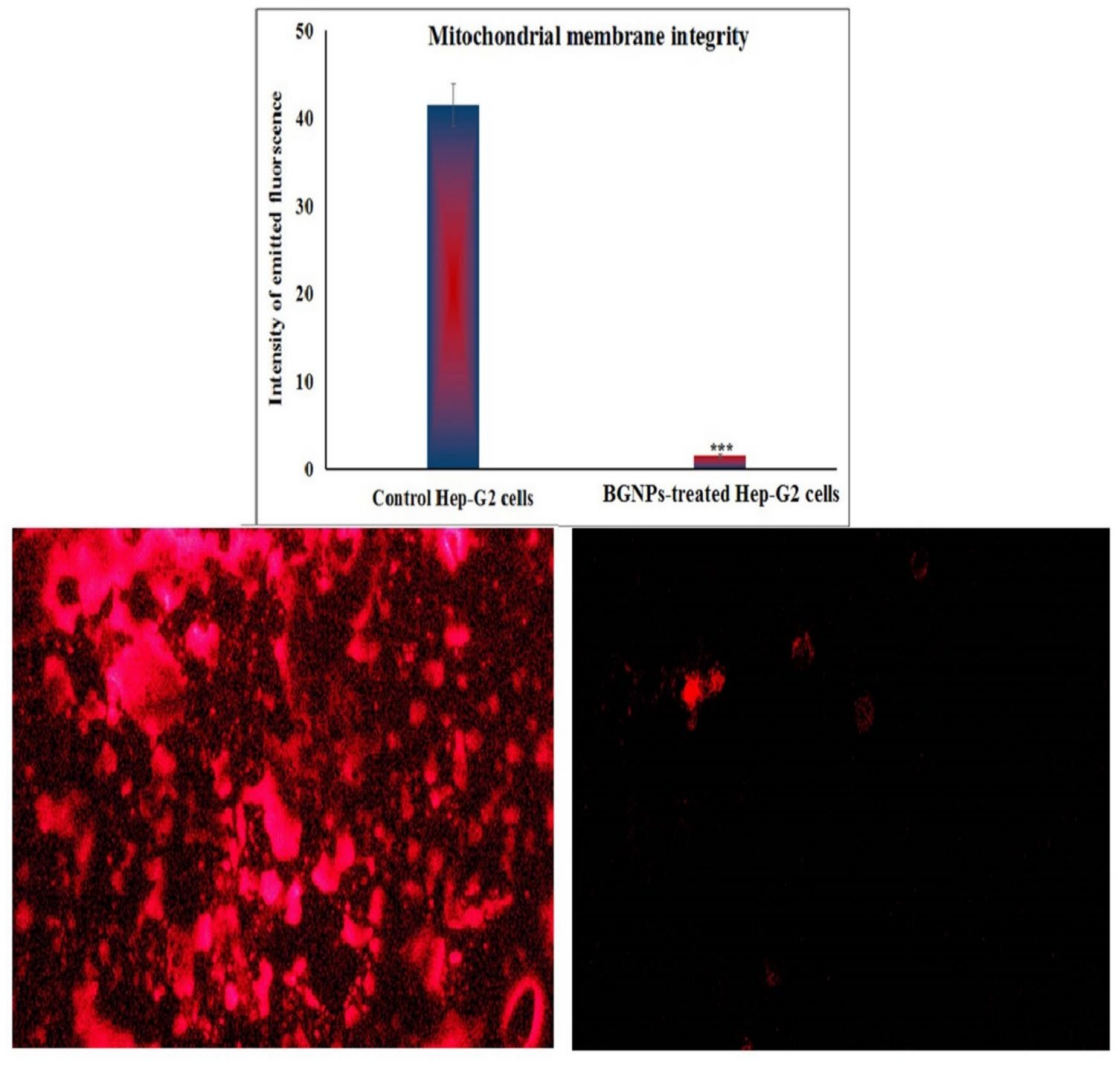
Integrity of mitochondrial membrane potential in the untreated and treated Hep-G2 hepatic cancer cells with the IC50 concentration (72.77 μg/mL) of BGNPs for 72 h. Magnification 200 ×

The impact of BGNPs on mitochondrial membrane potential in Hep-G2 hepatic cancer cells was assessed using Rhodamine-123, a fluorescent cationic dye that selectively accumulates in active mitochondria with intact membrane potential. Following 72 h of exposure to BGNPS at the IC50 concentration (72.77 μg/mL), a pronounced loss of mitochondrial membrane potential was observed. As depicted in Fig. 7, this dramatic disruption was evidenced by a statistically significant reduction (*p* < 0.001) in Rhodamine-123 fluorescence intensity in BGNP-treated Hep-G2 cancer cells compared to untreated control cells. The diminished fluorescence signal indicates a collapse of mitochondrial membrane potential, reflecting severe impairment of mitochondrial function and integrity in BGNP-treated Hep-G2 cells.

### BGNPs induce apoptotic cell death of Hep-G2 hepatic cancer cells

**Fig. 8:**
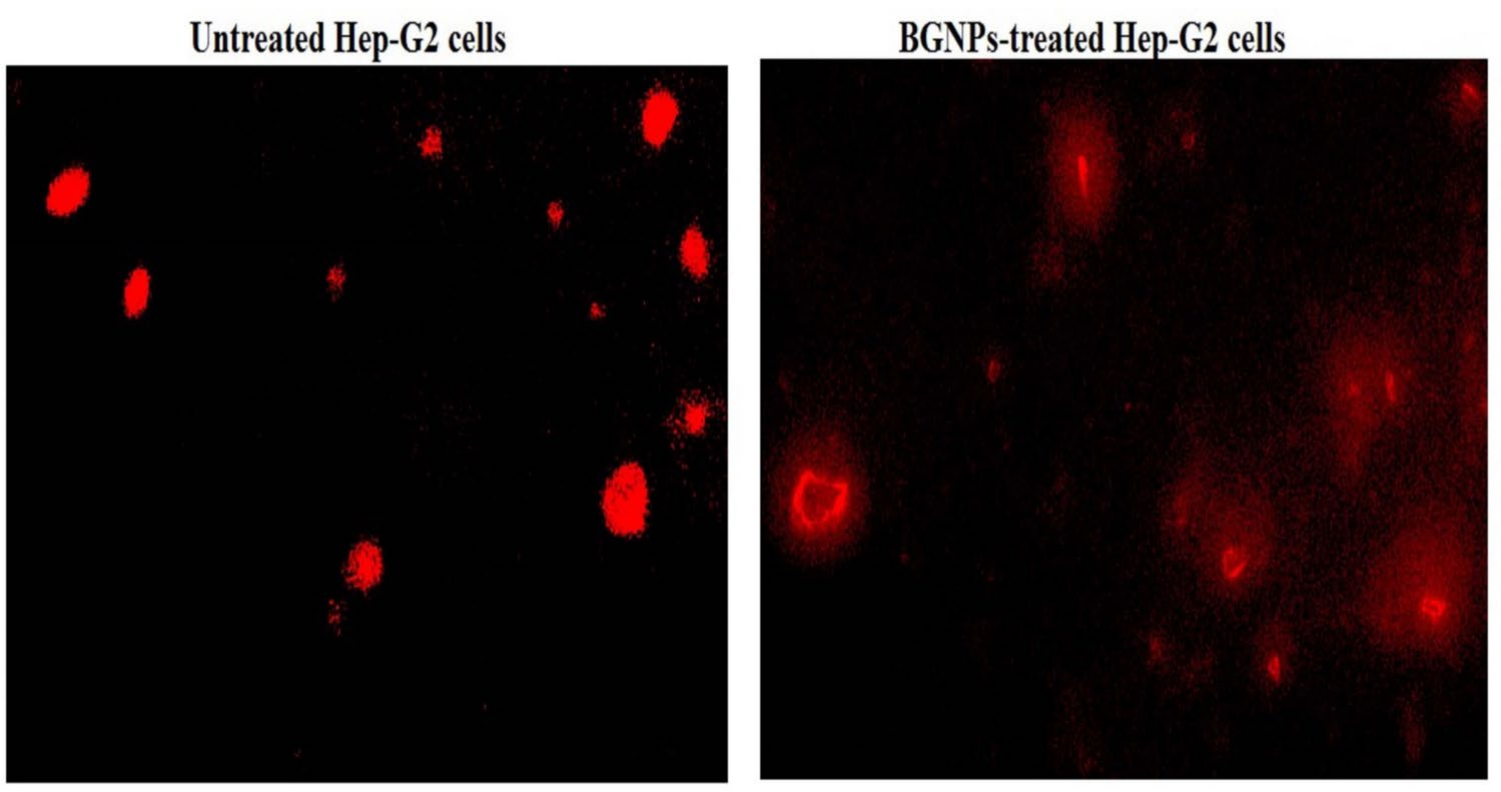
Chromatin diffusion assay distinguishing normal cells with intact DNA from apoptotic cells exhibiting diffused DNA, respectively, in untreated and BGNP-treated Hep-G2 hepatic cancer cells following 72-h exposure to the IC50 concentration (72.77 μg/mL) Magnification: 200 ×

**Fig. 9:**
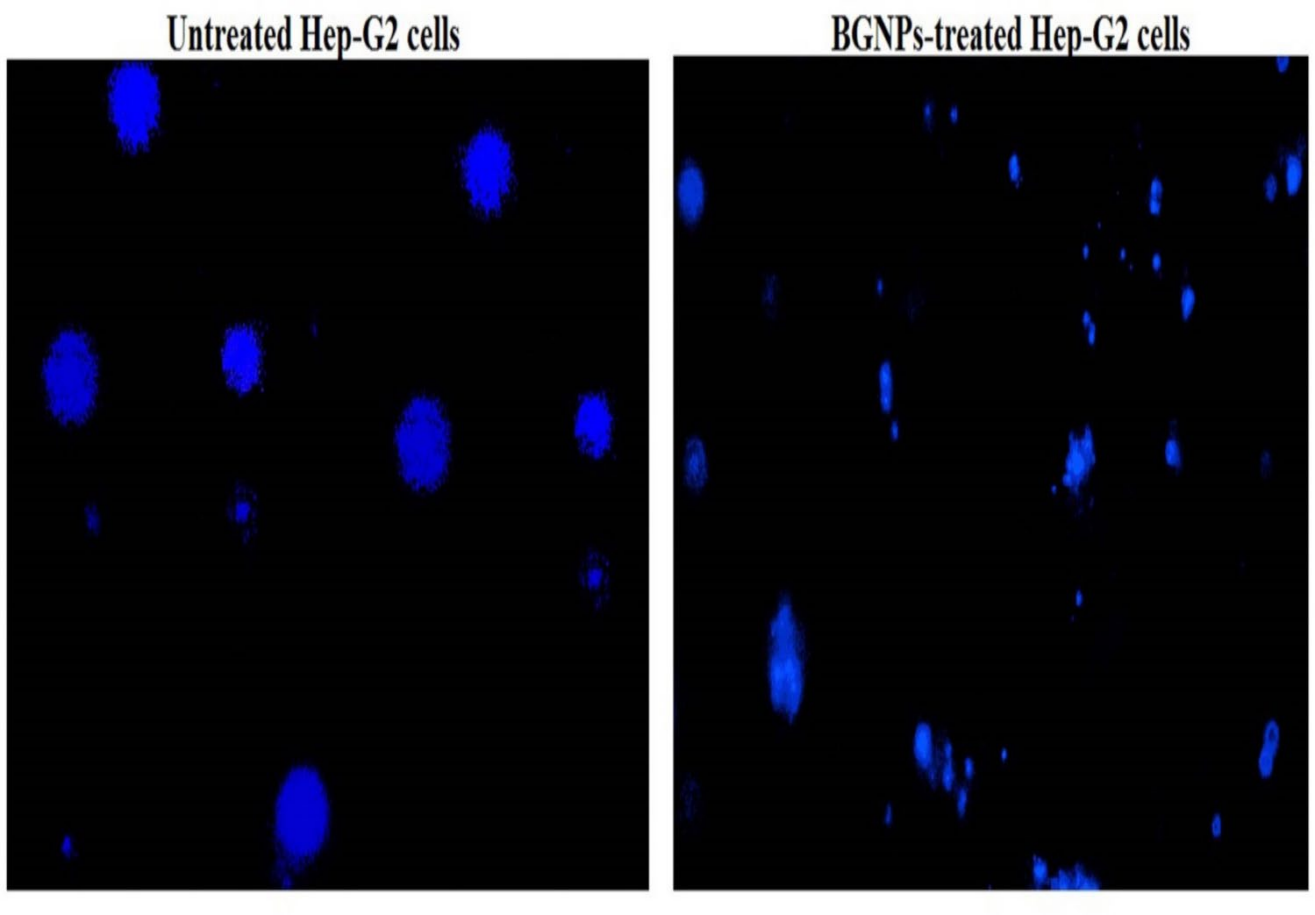
DAPI staining illustrating intact, uniformly stained nuclei in untreated Hep-G2 cells and condensed or fragmented nuclei in apoptotic Hep-G2 cells treated with the IC50 concentration (72.77 μg/mL) of BGNPs for 72 h. Magnification: 200 ×

The induction of apoptosis by BGNPs in Hep-G2 hepatic cancer cells was investigated using both the chromatin diffusion assay and DAPI staining. The combined findings from these assays provide strong evidence that exposure to BGNPs at the IC50 concentration for 72 h effectively induces apoptosis in Hep-G2 cells, as indicated by nuclear morphological alterations and chromatin fragmentation (Figs. 8 and 9).

In the chromatin diffusion assay, apoptotic cells were identified by the formation of characteristic chromatin diffusion halos, which result from the dispersion of fragmented DNA into the cytoplasm. Microscopic examination revealed that BGNP-treated Hep-G2 cells exhibited prominent chromatin halos surrounding condensed nuclei, in sharp contrast to the compact, intact nuclei seen in untreated control cells (Fig. 8). Quantitative analysis confirmed a significant increase (*p* < 0.001) in the number of cells displaying chromatin diffusion halos, as well as in the percentage of apoptotic Hep-G2 cells following BGNPs treatment (Table 3).

**Table 3:** Apoptosis detection via chromatin diffusion assay in Hep-G2 human hepatocellular carcinoma cells after 72-h exposure to the IC50 concentration (72.77 μg/mL) of BGNPs

	Treatment (concentration)	Cells with intact DNA	Cells with diffused DNA	% apoptotic cells
Hep-G2 cancer cells	Untreated (0.00 μg/mL)	938.00 ± 2.00	62.00 ± 2.00	6.2 ± 0.2
	BGNPs treated (72.77 μg/mL)	643.67 ± 6.11^***^	356.33 ± 6.11^***^	35.6 ± 6.11^***^

Results are expressed as mean ± SD

^***^Indicates statistical significant difference from the compared untreated Hep-G2 control cells at *p* < 0.001, using independent Student *t*-test

To further validate these findings, DAPI staining was performed. DAPI (4′,6-diamidino-2-phenylindole) binds specifically to DNA, allowing clear visualization of nuclear morphology associated with apoptosis. Fluorescence microscopy of control Hep-G2 cells showed uniformly stained, round nuclei with smooth outlines, indicative of viable cells (Fig. 9). In contrast, BGNP-treated cells displayed pronounced apoptotic features, including chromatin condensation, nuclear shrinkage, fragmentation, and the appearance of apoptotic bodies, visible as bright, condensed, and fragmented nuclei (Fig. 9). The percentage of apoptotic nuclei was significantly higher (*p* < 0.001) in BGNP-treated cells compared to untreated controls. Collectively, these results demonstrate that BGNPs induce apoptosis in Hep-G2 cancer cells through nuclear condensation and chromatin fragmentation, supporting their potential as pro-apoptotic agents in hepatic cancer therapy as shown in Table 4.

**Table 4:** Apoptosis detection via DAPI staining assay in Hep-G2 human hepatocellular carcinoma cells after 72-h exposure to the IC50 concentration (72.77 μg/mL) of BGNPs

	Treatment (concentration)	Intact cells	Apoptotic cells	% apoptotic cells
Hep-G2 cancer cells	Untreated (0.00 μg/mL)	928.33 ± 3.05	71.67 ± 3.05	7.17 ± 0.30
	BGNP treated (72.77 μg/mL)	540.33 ± 12.86^***^	459.67 ± 12.56^***^	45.97 ± 1.28^***^

Results are expressed as mean ± SD

^***^Indicates a statistical significant difference from the compared untreated Hep-G2 control cells at *p* < 0.001, using independent Student *t*-test

### BGNPs markedly disrupt p53, ND3, and Bcl2 gene expression in Hep-G2 cancer cells

The results of the qRT-PCR analysis, summarized in Table 5, demonstrate significant alterations in the expression of genes associated with apoptosis and mitochondrial function following treatment of Hep-G2 hepatic cancer cells with the IC50 concentration (72.77 μg/mL) of BGNPs for 72 h. The expression of the p53 apoptotic gene was significantly upregulated (*p* < 0.001) in BGNP-treated cells compared to untreated control cells suggesting activation of a p53-mediated apoptotic pathway in response to BGNPs exposure. In contrast, the expression level of both the ND3 mitochondrial gene and the Bcl-2 anti-apoptotic gene was significantly downregulated (*p* < 0.001) following Hep-G2 cell treatment with BGNPs. The reduction in ND3 expression indicates potential mitochondrial dysfunction, while the decreased Bcl-2 expression suggests a suppression of anti-apoptotic signaling mechanisms.

**Table 5:** Fold change in the expression level of p53, ND3, and Bcl2 genes in Hep-G2 human hepatocellular carcinoma cells after 72-h exposure to the IC50 concentration (72.77 μg/mL) of BGNPs

	Treatment (concentration)	Fold change in the expression of		
		p53 gene	ND3 gene	Bcl2 gene
Hep-G2 cancer cells	Untreated (0.00 μg/mL)	1.00 ± 0.00	1.00 ± 0.00	1.00 ± 0.00
	BGNP treated (72.77 μg/mL)	8.16 ± 0.88^***^	0.29 ± 0.03^***^	0.52 ± 0.02^***^

Results are expressed as mean ± SD

^***^Indicates statistical significant difference from the compared untreated Hep-G2 control cells at *p* < 0.001, using independent Student *t*-test

## Discussion

Liver cancer, particularly HCC, remains a major global health burden due to its high mortality rate, late-stage diagnosis, and the limited efficacy of current therapeutic options. While chemotherapeutic agents remain a cornerstone of HCC treatment, their clinical use is often restricted by severe systemic side effects including hepatotoxicity, nephrotoxicity, and hematological toxicity, underscoring the urgent need for safer and more targeted therapeutic alternatives (Samant et al. 2021; Addissouky et al. 2024). In recent years, nanoparticle-based approaches have garnered increasing attention for their potential to improve treatment specificity and reduce off-target toxicity. Among these, BGNPs have been extensively studied for their biocompatibility, bioactivity, and capacity to release therapeutic ions that can modulate cellular processes, especially in the fields of bone regeneration and antimicrobial therapy (Hoppe et al. 2011; Jones 2013; Pajares-Chamorro and Chatzistavrou; 2020; Drevet et al. 2024). However, their anticancer potential remains largely unexplored, particularly in the context of HCC.

**Fig. 10:**
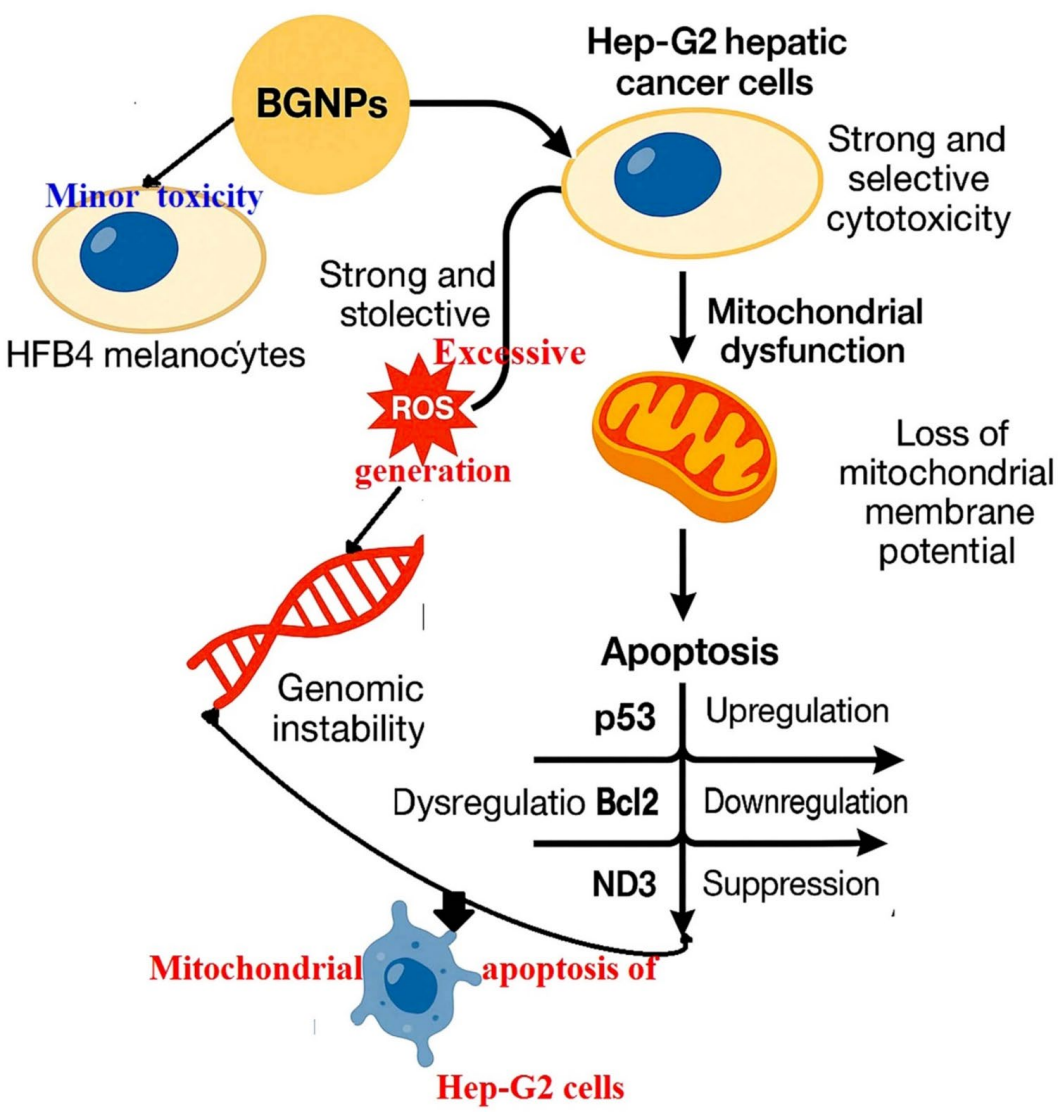
Mechanistic pathway of BGNPs induced selective and potent cytotoxicity in Hep-G2 hepatic cancer cells

In light of the growing need for safer and more effective cancer therapies, the present study was designed to estimate the cytotoxicity of BGNPs on both normal human HFB4 melanocytes and Hep-G2 hepatic cancer cells. In addition, the potential of BGNPs to induce DNA damage, mitochondrial dysfunction, oxidative stress, and apoptosis was investigated in Hep-G2 cells, thereby uncovering the molecular mechanisms underlying their potential anticancer activity. This investigation provides novel insights into the anticancer potential of BGNPs in HCC models, supporting the growing body of evidence on the repurposing of inorganic nanoparticles as novel anticancer agents, particularly for malignancies such as HCC, which have limited therapeutic options. As illustrated in Fig. 10, BGNPs exhibited strong, selective cytotoxic, and pro-apoptotic effects on cancer cells while sparing normal cells. These effects were accompanied by the upregulation of the pro-apoptotic gene p53 and downregulation of the anti-apoptotic gene Bcl-2 and mitochondrial gene ND3, suggesting that BGNPs mediate Hep-G2 cancer cell death through mitochondrial impairment and transcriptional regulation of apoptosis-related pathways. Altogether, these findings position BGNPs as promising candidates for future preclinical and clinical exploration in the context of liver cancer treatment. The specific experimental outcomes and mechanistic interpretations are discussed in detail in the subsequent points.

The characterization results confirmed that the BGNPs possess key physicochemical properties suitable for biomedical applications. The broad halo in the XRD pattern indicated their amorphous structure, an advantageous feature that enhances the reactivity of the glass matrix by facilitating ion exchange and surface activity (Vecchio et al. 2024). The low polydispersity index (PDI < 0.3), detected by DLS analysis, indicates a uniform size distribution and stable colloidal behavior, essential for consistent biological interactions and dispersion in aqueous media (Danaei et al. 2018). TEM imaging further supported these findings by revealing spherical nanoparticles with minimal aggregation and consistent morphology. The slightly larger particle sizes observed via DLS, compared to TEM, are attributed to the measurement of hydrodynamic diameter, which includes the solid core, hydration shell, and minor agglomerates, unlike TEM which visualizes only the dehydrated core (Liang et al. 2015; Wilson and Prud’homme 2021). Despite this difference, the consistently low PDI underscores a narrow size distribution and reinforces the stability and reproducibility of the nanoparticles, supporting their potential for future biomedical use.

From a biological perspective, the results of MTT cytotoxicity assay provide strong evidence for the selective and potent anticancer potential of BGNPs against Hep-G2 hepatic cancer cells. Treatment with two-fold increasing concentrations of BGNPs (ranging from 7.8 to 1000 μg/mL) led to a marked, concentration-dependent reduction in Hep-G2 cell viability, with an IC50 value of 72.77 μg/m, indicating substantial sensitivity of these cancer cells to BGNPs. In contrast, normal HFB4 melanocytes exhibited only minimal cytotoxic responses under the same treatment conditions, with a significantly higher IC50 value of 360.4 μg/mL. This marked difference in cytotoxic response is further emphasized by a high selectivity index (SI) of 4.95, underscoring the ability of BGNPs to selectively target malignant Hep-G2 cells while largely sparing normal cells. Such selective cytotoxicity aligns well with the therapeutic goal of minimizing off-target effects typically associated with conventional chemotherapy. Notably, SI values above 2 are considered indicative of effective selectivity, reinforcing the potential of BGNPs as a safer and more targeted therapeutic approach for liver cancer. These findings are consistent with recent studies by Fellenberg et al. (2022) and Deliormanlı et al. (2024), which demonstrated strong cytotoxic effects of macroparticulate bioactive glass on osteosarcoma (SaOS-2) and giant cell tumor cells, alongside minimal toxicity to normal bone-derived stromal cells, further supporting the potential of bioactive glass-based materials in selective cancer therapy.

The precise mechanisms through which BGNPs exert selective cytotoxicity in Hep-G2 cancer cells are not yet fully understood. To gain deeper insight into BGNP-induced cytotoxicity, further mechanistic studies were conducted, including evaluation of DNA integrity, ROS generation, mitochondrial membrane potential, and apoptosis induction. Genomic instability, an established hallmark of cancer therapy-induced cytotoxicity, was prominently observed in Hep-G2 cells treated with BGNPs at their IC50 concentration. This genomic destabilization was clearly demonstrated by the alkaline single-cell gel electrophoresis (Comet) assay, which showed significant increases in tail length, %DNA in the tail, and tail moment. These metrics are well-recognized indicators of DNA strand breaks and alkali-labile sites (Collins 2004; Mohamed et al. 2025a, b, c; Mohamed et al. 2025a), suggesting that BGNPS exposure leads to pronounced nuclear DNA damage. Such damage plays a pivotal role in activating downstream apoptotic pathways, ultimately contributing to cancer cell death.

The genomic DNA disruption observed in Hep-G2 cells is likely mediated by oxidative stress, a well-documented consequence of nanoparticle exposure. BGNPs can generate ROS due to their large surface area and highly reactive silicate-based composition (Hench and Polak 2002; Rahaman et al. 2011). Elevated ROS levels can cause various forms of DNA damage, including base modifications, single- and double-strand breaks, and DNA–protein crosslinks (Klaunig et al. 2011). Cancer cells such as Hep-G2 often possess impaired antioxidant defense systems, rendering them more susceptible to ROS-induced DNA damage (Trachootham et al. 2009). In this context, the pronounced selective cytotoxicity observed in Hep-G2 cells following BGNPs exposure is likely due to ROS-mediated DNA damage that overwhelms the redox balance and DNA repair capacity of Hep-G2 cells, ultimately triggering apoptotic cell death.

Furthermore, ROS generated by BGNPs can compromise mitochondrial DNA integrity and disrupt nuclear chromatin structure, activating DNA damage response (DDR) pathways. Persistent or extensive unrepairable DNA damage can result in activation of cell cycle checkpoints, leading to growth arrest, senescence, or apoptosis (Nel et al. 2006; Jackson and Bartek 2009). This response is particularly pronounced in cancer cells with defective DDR components, such as mutations in tumor suppressors like p53, which diminish their capacity to manage genotoxic stress (Vousden and Lane 2007). In Hep-G2 cancer cells, the extent of BGNP-induced DNA damage may suppress the repair capacity, ultimately leading to cell death.

In addition, BGNPs may disrupt DNA replication and repair processes by directly interacting with key enzymes or through the release of ions such as calcium and phosphate, which can alter chromatin organization, nuclear signaling pathways, and enzyme activity (Chen et al. 2024; Ren et al. 2025). This could further compromise genomic stability and promote apoptosis. Notably, similar genotoxic effects have been reported in osteosarcoma and glioma models exposed to bioactive glass and related nanomaterials, supporting the broader applicability of BGNPs as a selective anticancer agent across various tumor types (Hoppe et al. 2011; Jones 2013).

Apoptotic cell death was confirmed in BGNPS-treated Hep-G2 cells through nuclear morphological analysis using DAPI staining and chromatin diffusion assays. Treated Hep-G2 cells exhibited classical features of apoptosis, including chromatin condensation, nuclear shrinkage, nuclear fragmentation, and the formation of apoptotic bodies. These morphological changes were accompanied by a marked loss of mitochondrial membrane potential, as detected by diminished Rhodamine-123 fluorescence. The disruption of mitochondrial membrane potential is a key event in the intrinsic (mitochondrial) apoptotic pathway, indicating that mitochondrial dysfunction is central to BGNPS-induced apoptosis.

Apoptotic cell death in BGNPS-treated Hep-G2 cells was confirmed through nuclear morphological analysis using DAPI staining and chromatin diffusion assays. Treated cells exhibited hallmark features of apoptosis, including chromatin condensation, nuclear shrinkage, nuclear fragmentation, and the formation of apoptotic bodies, consistent with previously described apoptotic phenotypes (Elmore 2007; Mohamed et al. 2025b). These morphological changes were accompanied by a significant loss of mitochondrial membrane potential, as detected by diminished Rhodamine-123 fluorescence, a key indicator of mitochondrial dysfunction. The loss of mitochondrial membrane potential is a pivotal event in the intrinsic (mitochondrial) apoptotic pathway, suggesting that mitochondrial impairment is central to BGNP-induced apoptosis (Green and Kroemer 2004).

Supporting this finding, qRT-PCR analysis revealed significant alterations in the expression of genes related to apoptosis and mitochondrial function, reinforcing the role of mitochondrial-mediated pathways in BGNP-induced cytotoxicity. Notably, treatment with BGNPs at the IC50 concentration led to a substantial upregulation of p53 gene, a key transcription factor involved in DNA damage response and apoptosis. Concurrently, there was a pronounced downregulation of Bcl-2, an anti-apoptotic gene that preserves mitochondrial integrity by inhibiting cytochrome c release. Additionally, the mitochondrial gene ND3, which encodes a subunit of Complex I in the electron transport chain, was significantly suppressed, indicating compromised mitochondrial respiration and ATP production. This combination of increased pro-apoptotic signaling, suppressed anti-apoptotic defense, and impaired mitochondrial function suggests a multifaceted mechanism of BGNPs-induced apoptosis. Consistency with previous studies (Cory and Adams 2002; Vousden and Lane 2007; Guan and Guan 2020; Mohamed et al. 2024), these findings overall indicate that BGNPs selectively induce cytotoxicity in Hep-G2 cells through a synergistic mechanism involving ROS generation, DNA damage, mitochondrial disruption, and transcriptional reprogramming of apoptotic pathways.

Taken together, this study provides the first evidence that BGNPs exert direct cytotoxic effects against hepatocellular carcinoma Hep-G2 cells in the absence of chemotherapeutic drug loading. In contrast to prior research that has mainly focused on their applications in regenerative medicine or as drug delivery carriers, our findings highlight the intrinsic anticancer potential of BGNPs, mediated through a multifaceted mechanism involving ROS-driven oxidative stress, mitochondrial dysfunction, genomic instability, and apoptosis induction. Importantly, BGNPs demonstrated selective toxicity toward Hep-G2 cells with minimal impact on normal HFB4 melanocytes, suggesting a favorable therapeutic index and supporting their promise as standalone nanotherapeutic candidates for liver cancer. Moreover, the mechanistic insights into the ROS–mitochondria–DNA damage axis enrich the current understanding of how BGNPs may be exploited in oncology. Nonetheless, certain limitations should be acknowledged. The experiments were confined to in vitro settings, which may not fully capture the complexity of the tumor microenvironment in vivo. While ROS generation, mitochondrial impairment, and gene expression alterations were investigated, additional molecular validation; such as γ-H2AX foci detection, transcriptomic or proteomic profiling, and pathway-specific inhibition assays, would further substantiate the mechanistic conclusions. The reliance on a single cancer cell line and one normal cell type also constrains the generalizability of the findings. Finally, issues of pharmacokinetics, biodistribution, and long-term biosafety remain unaddressed, underscoring the need for future studies employing normal hepatocyte models and in vivo validation to establish the translational potential of BGNPs for liver cancer therapy.

## Conclusion

Collectively, the results of this study demonstrate that BGNPs exert potent and targeted anticancer effect against Hep-G2 hepatic cancer cells by disrupting genomic integrity and promoting apoptosis. This is mediated through a multifaceted mechanism involving ROS-mediated mitochondrial damage and the dysregulation of genes involved in apoptosis and mitochondrial function. These findings provide valuable insights into the molecular actions of BGNPs and highlight their promise as a novel nanotherapeutic platform for hepatocellular carcinoma, with possibly lower toxicity than traditional chemotherapy. To strengthen these findings, further investigations using γ-H2AX foci formation, Comet-FISH, and transcriptomic profiling of DNA repair pathways are recommended to directly assess genomic instability and DNA repair engagement. In vivo studies and targeted delivery approaches are also crucial to validate and enhance the therapeutic potential of and the clinical relevance of BGNPs for liver cancer therapy.

## Data Availability

The datasets used and/or analyzed during the current study are available from the corresponding author on reasonable request.
